# Is Swine-origin Influenza a Predisposing Factor for Deep Vein Thrombosis?

**DOI:** 10.5505/tjh.2012.25932

**Published:** 2012-06-15

**Authors:** Müge Gökçe, Şule Ünal, Selin Aytaç, Ateş Kara, Mehmet Ceyhan, Murat Tuncer, Fatma Gümrük

**Affiliations:** 1 Hacettepe University, School of Medicine, Department of Pediatric Hematology, Ankara, Turkey; 2 Hacettepe University, School of Medicine, Department of Pediatric Infectious Disease, Ankara, Turkey

**Keywords:** children, Deep venous thrombosis, Swine-origin (pandemic) influenza

## Abstract

Herein we report a sixteen-year-old female that developed deep vein thrombosis (DVT) while undergoing treatment for H1N1 pneumonia. To the best of our knowledge this is the first report of H1N1/09 infection complicated by DVT in an adolescent patient with no detected risk factors other than immobilization. Healthcare providers should be aware of the possibility of thrombosis in patients with swine-origin influenza, especially in those with additional risk factors.

## Abstract (tr)

Burada H1N1 enfeksiyonu için tedavi alırken, derin ven trombozu geliştiren bir olguyu sunmaktayız. Mevcut bilgilerimize gore; H1N1 enfeksiyonu sırasında - immobilizasyon dışında başka hiçbir risk faktörü olmayan - bir adölesanda tromboz gelişimi ilk defa bildirilmektedir. Domuz gribi pandemilerinde özellikle eşlik eden risk faktörleri olanlarda tromboz gelişebileceği akılda tutulmalıdır.

## INTRODUCTION

Venous thromboembolism (VTE) is a multifactorial entity that arises due to variable interactions between genetic and environmental components, with an incidence among children of 0.07-0.14/10,000 [1]. The incidence of VTE in hospitalized children has been reported to be as high as 58 cases per 10,000 hospital admissions [2]. The majority of children with VTE have comorbid medical conditions. Infection, dehydration, trauma, cancer, central venous catheterization, and chemotherapy are the most common predisposing factors for the development of thrombosis in children [3]. We reported earlier that thrombosis was associated with infection in 68% of hospitalized children with thrombosis [4].

Since April 2009, H1N1 infection has been a major concern, as it has spread rapidly worldwide. Herein, we report a sixteen-year-old female that developed thrombosis in the deep femoral vein while undergoing treatment for H1N1 pneumonia.

## CASE

A sixteen-year-old female was admitted to the hospital due to fever (41 °C), difficulty breathing, and perioral cyanosis. She had been prescribed oseltamivir phosphate (Tamiflu) for pandemic influenza A. She has cerebral palsy due to periventricular leukomalacia as a complication of premature birth, and had undergone surgery for developmental dysplasia of the left hip without thrombotic complication at age 3 years. Written informed consent was obtained from the patient. 

Physical examination showed tachypnea, dyspnea, and perioral cyanosis. Oxygen saturation was 57% based on pulse oximetry. Bilateral inspiratory crackles were present on auscultation. Because of her neurological condition, the arms were fully flexed, whereas her legs were extended, with no apparent difference in diameters. Deep tendon reflexes were hyperactive, the plantar response was extensor, and the remainder of the physical examination was unremarkable. 

Arterial blood gas analysis indicated respiratory acidosis based on the following results: pH: 7.08; pCO_2_: 59.8 mmHg; pO_2_: 61.9 mmHg. Complete blood count was as follows: hemoglobin: 10 g dL^–1^; hematocrit: 31.2%; WBC count: 8.3 x 10^9^ L^–1^; thrombocyte count: 318 x 10^9^ L^–1^. Liver and kidney function test results were within normal limits. Chest X-ray showed bilateral reticular infiltration. Nasopharyngeal swab for pandemic influenza was taken. Oral clarithromycin and parenteral clindamycin were added to her treatment. With oxygen support and bronchodilation treatment, the hypoxemia subsided. Polymerase chain reaction analysis for influenza virus H1N1 was positive [5]. The patient did not require mechanical ventilation support and her respiratory status improved during the next few days. 

On the fifth day of the treatment her left leg appeared to be swollen and larger in diameter than the right leg. Doppler ultrasonography showed a hyperechogenic thrombus with non-compressibility in the left deep femoral vein. Enoxaparin 1 mg kg^–1^ b.i.d. was initiated and detailed prothrombotic risk factor analysis was performed. Basal activated partial thromboplastin time (aPTT) and international normalized ratio (INR) were within normal limits. The plasma fibrinogen level was 436 mg dL^–1^ (normal range: 200-450 mg dL^–1^) and thrombin time was 14 s (normal range: 12-16 s). D-dimer was 5.42 μg mL^–1^ (normal range: 0-0.48 μg mL^–1^), anti-thrombin III activity was 101% (normal range: 80%-120%), plasma protein C and protein S levels were 84% (normal range: 70%-130%) and 92% (normal range:60%-130%), respectively, and the plasma factor VIII level was 126% (normal range: 53%- 170%). Anticardiolipin and antiphospholipid antibodies were negative. The serum lipid profile, and lipoprotein-a and homocysteine levels were normal. Analysis for methylenetetrahydrofolate reductase (MTHFR C677T –/–), factor V Leiden, and prothrombin 20210A mutations were negative. Echocardiographic evaluation showed normal systolic and diastolic functions without intracardiac thrombusthrombus. Thoracic CT performed to investigate an associated pulmonary thromboembolism (PTE) was unremarkable. Swelling in the patient’s left leg regressed by the 3^rd^ day of enoxaparin treatment, and she was discharged 10 d after admission with a therapeutic antiXa level.

## DISCUSSION

The pathophysiology of thrombosis is based on the presence of 3 factors: endothelial injury, hypercoagulability, and venous stasis. Damage to vessel walls prevents endothelium from inhibiting coagulation and initiating local fibrinolysis. As VTE is usually multifactorial in childhood, detailed analysis should be performed in an effort to determine the underlying etiology. 

In addition to coagulation cascade activation, inflammatory response plays a role in thrombus formation via production of microparticles (MPs) that carry cell-specific molecules—proteins such as galectins [6]. Stimulation of monocytes, especially by galectin-1, leads to cell activation and tissue factor expression that triggers the coagulation pathway. Furthermore, oxygen free radicals produced during inflammation are known to induce thrombosis in microvessels via activation of platelet aggregation [7]. Infectious agents, including bacteria, viruses, and parasites, may initiate this process. A 13-year-old male that developed deep vein thrombosis (DVT) and pulmonary embolism following pneumonia caused by Mycoplasma pneumoniae was reported; however, protein S deficiency and transient lupus anticoagulant were underlying risk factors [8]. It is also known that varicella zoster infection can cause thrombosis in patients with protein S and C deficiency [9]. An 8-year-old boy that developed middle cerebral artery thrombosis as a result of Streptococcus oralis infection was reported [10]. 

Since being identified in Mexico City in April 2009, pandemic influenza A (H1N1) has become a significant cause of morbidity and mortality worldwide. Till 6 December 2009, 208 countries have reported laboratory-confirmed cases of pandemic influenza H1N1 to the World Health Organization (WHO) [11]. Although hospitalized children with swine-origin influenza infection are more likely to have underlying medical conditions, healthy individuals are also at risk f H1N1 infection and infection-related death. Unusual clinical presentations of H1N1 infection reported to date have included parotitis, conjunctivitis, and extensive bowel involvement with mesenteric thrombosis and hemophagocytic syndrome [12,13]. 

The presented case developed DVT while undergoing treatment for H1N1 pneumonia. The patient did not have any coagulation abnormality or prothrombotic risk factor other than immobilization. The patient’s history of thrombotic episodes was negative despite having cerebral palsy and having undergone surgery for hip dysplasia. The patient’s family history of thrombosis was also negative. Detailed thrombotic risk factor analysis was performed to clarify the association between swine flu and DVT. H1N1 infection in the presented case might have triggered the formation of thrombosis because of endothelial injury in the left deep femoral vein due to previous surgery. D-dimer elevation indicated acute thrombosis. Harms et al. performed autopsy on 8 patients with H1N1 infection and reported that 5 had peripheral pulmonary vascular thrombosis and all 8 had cytophagocytosis [14]. An in vitro study reported that monocytes and endothelial cells that were incubated with influenza were able to activate coagulation via endothelial dysfunction and elevated tissue factor levels [15,16]. It is known that seasonal flu vaccine reduces the risk of cardiovascular events in patients with coronary heart disease. A novel study reported that influenza vaccination is associated with a reduced risk of VTE, whereas influenza infection itself may predispose individuals to thrombus formation, especially DVT [17]. 

In conclusion, thrombotic events should be considered among the possible complications of H1N1 infection, and low molecular weight heparin prophylaxis might be considered in immobilized patients with H1N1 infection. 

**Acknowledgements**

Each author contributed to this report equally. 

**Conflict of Interest Statement **

The authors of this paper have no conflicts of interest, including specific financial interests, relationships, and/or affiliations relevant to the subject matter or materials included.
